# Acupuncture for Preventing Complications after Radical Hysterectomy: A Randomized Controlled Clinical Trial

**DOI:** 10.1155/2014/802134

**Published:** 2014-04-15

**Authors:** Wei-min Yi, Qing Chen, Chang-hao Liu, Jia-yun Hou, Liu-dan Chen, Wei-kang Wu

**Affiliations:** ^1^Department of Traditional Chinese Medicine and Acupuncture, Sun Yat-sen Memorial Hospital of Sun Yat-sen University, Guangzhou 510120, China; ^2^Department of Traditional Chinese Medicine, The Third Affiliated Hospital of Sun Yat-sen University, Guangzhou 510635, China; ^3^Department of Gynecology Oncology, Sun Yat-sen Memorial Hospital of Sun Yat-sen University, Guangzhou 510120, China; ^4^Institute of Integrated Traditional Chinese and Western Medicine, Sun Yat-sen University, Guangzhou 510080, China

## Abstract

We aimed to investigate the preventive effects of acupuncture for complications after radical hysterectomy. A single-center randomized controlled single-blinded trial was performed in a western-style hospital in China. One hundred and twenty patients after radical hysterectomy were randomly allocated to two groups and started acupuncture from sixth postoperative day for five consecutive days. Sanyinjiao (SP6), Shuidao (ST28), and Epangxian III (MS4) were selected with electrical stimulation and Zusanli (ST36) without electrical stimulation for thirty minutes in treatment group. Binao (LI14) was selected as sham acupuncture point without any stimulation in control group. The main outcome measures were bladder function and prevalence of postoperative complications. Compared with control group, treatment group reported significantly improved bladder function in terms of maximal cystometric capacity, first voiding desire, maximal flow rate, residual urine, and bladder compliance, and decreased bladder sensory loss, incontinence, and urinary retention on fifteenth and thirtieth postoperative days. Treatment group showed significant advantage in reduction of urinary tract infection on thirtieth postoperative day. But no significant difference between groups was observed for lymphocyst formation. By improving postoperative bladder function, early intervention of acupuncture may provide a valuable alternative method to prevent bladder dysfunctional disorders and urinary tract infection after radical hysterectomy.

## 1. Background 

Cervical cancer, with an estimated 500,000 new cases diagnosed worldwide annually [1], ranks second in women's malignant diseases. Accounted for one-third of the world's total annual new cases, 83.9% of cervical cancer in China accepted surgical treatment [2]. The situation of high surgical rate is similar for most patients worldwide in the past decade [2–4]. Radical hysterectomy (RH) with pelvic lymphadenectomy is the mainstay of surgical treatment for early stage cervical cancer and stage II endometrial cancer according to the guideline version 2011 of National Comprehensive Cancer Network [5, 6].

The procedure of RH is associated with significant perioperative morbidity, following with some unavoidable complications. Bladder dysfunction (including sensory loss, incontinence, and urinary retention), lymphocyst formation, pelvic discomfort, ureterovaginal fistula, intestinal obstruction, and thromboembolic disease are the most common complications according to clinical practice and literatures [1, 7]. Surgeons and other doctors try to reduce complications by careful postoperative management. Thromboprophylaxis with heparin and lower extremity sequential compression devices considerably decrease the incidence of thromboembolic disease following RH. Preservation of the superior vesical artery and blood supply to the distal ureter also decrease vesicovaginal and ureterovaginal fistula after RH [1]. The nodus and controversy are located on bladder dysfunction and lymphocyst formation. 30%–85% of patients after RH are reported to have long-term postoperative urinary tract dysfunction [8, 9]. The severity of bladder dysfunction depends on radicality of the procedure, while the extent of lymphadenectomy has an impact on variation of lymphocyst formation. Prolonged bladder drainage allows time for repair and adaptation following injury to autonomic innervation. But it may increase the urinary tract infection and fibrosis. Although it may reduce damage to dominated sensory or motor nerves, laparoscopic nerve sparing RH has limited indications and may increase the possibility of uncontrollable tumor [10].

Complementary and alternative medicine, especially acupuncture, is commonly applied to prevent and treat complications after abdominal surgical procedure in Chinese hospitals. But few literatures are randomized controlled trial and published in English. In our previous study, acupuncture showed some effects in treating urinary retention after RH [11]. Refractory cases of urinary retention still occurred when we started acupuncture treatment two weeks after RH procedure.

In order to evaluate the preventive effects of acupuncture for complications after RH, we shifted forward postoperative acupuncture intervention in current study. Not only bladder dysfunction was observed, but other complications, such as lymphocyst formation, urinary tract infection, and wound infection, were also paid attention to.

## 2. Methods

### 2.1. Design

This was a single-center, prospective, single-blinded, parallel-group, randomized controlled trial. The trial protocol strictly followed the principles of the Declaration of Helsinki (version 2008) and approval had been obtained from the Ethical Committee of Sun Yat-sen Memorial Hospital, Sun Yat-sen University. All participants were required to give written informed consent.

### 2.2. Participants

Patients were recruited from Sun Yat-sen Memorial Hospital of Sun Yat-sen University (SYSMH) from May 2012 to December 2013. The inclusion criteria were (1) schedule for Piver III radical hysterectomy and pelvic lymphadenectomy without nerve-sparing, no history of hypogastric operation; (2) age between 20 and 65 years old; (3) normal liver and kidney function and normal ECG on preoperative test; (4) no experience with acupuncture therapies; and (5) willingness to participate and signed an informed consent form.

The exclusion criteria were (1) serious systemic or neurologic disease (diabetes, AIDS, epilepsy, etc.); (2) paruria, urinary system infection, or calculus on preoperative test; (3) preoperative radiotherapy or chemotherapy; (4) cardiac pacemaker; or (5) refusal to accept acupuncture treatment.

### 2.3. Randomization

The computerized randomization scheme was designed by Medical Statistical Teaching and Research Section of Sun Yat-sen University. Using a 1 : 1 treatment ratio, there were 120 women assigned to each group. The randomization sequence was generated with a block of 6. The numbers of screening sequence were printed on the surface of envelop, while the group names were put inside. The patients and statistician were blinded to the group assignments, but the acupuncturists were not blinded due to the procedure. The patients did not know each other and would be separated to different rooms once they were scheduled to perform radical hysterectomy. If there were more than or equal to two patients in one day, they were required to reach acupuncture clinics at different times during the day.

### 2.4. Intervention

Both treatment and control groups accepted regular postoperative medication and nursing care. Two accredited acupuncturists had more than 8 years of acupuncture experience and clinical research training. The acupuncture intervention started from the 6th postoperative day, once a day and for five consecutive days. Patients lay in a supine position when they were accepting treatment.

#### 2.4.1. Treatment Group

Acupoints of Sanyinjiao (SP6), Zusanli (ST36), Shuidao (ST28), and Epangxian III (MS4) were selected bilaterally according to WHO standard acupuncture point locations. Stainless steel needles (0.30 × 25 mm, Tianxie, Suzhou, China) were inserted to the acupoints with relevant depth and direction after 75% alcohol prepped on the skin. SP6 and ST36 were punctured at a depth of 20 mm vertically. ST28 was punctured 20 mm obliquely towards symphysis pubica. MS4 was inserted 15 mm horizontally. Needles were manipulated to achieve “De Qi,” a sensation of numbness, distention, soreness, and heaviness on the acupoints [12]. Electrical stimulator (G6805-I, Xinsheng, Qingdao, China) was put on the needles of bilateral SP6, ST28, and MS4 concomitantly and continuously for 30 minutes, with appropriate strength of slight vibration reported by patients. Continuous wave was selected with a frequency of 4 Hz.

#### 2.4.2. Control Group

Binao (LI14) was selected bilaterally with superficial needle insertion less than 3 mm. The leads of electrical stimulator were put on LI14 for 30 minutes without any manual or electrical stimulation.

LI14 was commonly used as sham acupoints and for local disease [13]. It did not have specific effects on lower abdominal diseases or symptoms according to literatures and clinical experiences. Patients in this group provided a baseline evaluation of postoperative complications in the study.

### 2.5. Outcome Measures

#### 2.5.1. Bladder Function

Urodynamic examination was checked on 15th and 30th postoperative days. It consisted of five items, including first voiding desire, maximal cystometric capacity, maximal flow rate, postvoided residual urine, and bladder compliance. All urethral catheters were removed after urodynamic examination on the 15th postoperative day. Patients were encouraged to void after catheter removal. If the postvoided residual (PVR) urine volume was greater than 100 mL on 15th postoperative day, urinary retention after RH would be diagnosed and catheter would be retained. Then taking seven days as an interval, PVR was checked again to access the necessity of prolonged catheterization until PVR was less than 100 mL. Sensory loss was defined as bladder volume more than 200 mL when first voiding desire appeared.

#### 2.5.2. Postoperative Complications

The prevalence of sensory loss, incontinence, and urinary retention was assessed by urodynamic examination and patients' reports together on the 15th and 30th postoperative days. Lymphocyst formation was checked by color Doppler ultrasound on the 15th day and 30th postoperative days. The occurrence of lymphedema, ileus, ureterovaginal fistula, and wound infection was recorded by gynecologists. Lymphedema and ureterovaginal fistula were diagnosed by clinical manifestation and color Doppler ultrasound. Ileus was evaluated by clinical manifestation and X-ray. Wound infection would be diagnosed by testing cultivation of anaerobic and aerobic bacteria from abnormal wound effusion. The observation period included two stages, from the 6th postoperative day to the 15th postoperative day and from the 16th postoperative day to the 30th postoperative day. Urine routine examination would be checked on 15th day and 30th day in order to evaluate the condition of urinary tract infection. If the leukocyte in medistream urine was positive, the diagnosis of urinary tract infection would be established.

#### 2.5.3. Adverse Events

The adverse events related to the acupuncture treatment reported during the research were assessed by the Common Terminology Criteria for Adverse Events (CTCAE) version 4.0 [14].

### 2.6. Statistical Analysis

All data were analyzed using the Statistical Package for the Social Sciences (SPSS) version 18.0 statistics software by a blinded statistician at a separate location. The carry-forward principle of intention-to-treat was used to analyze the data. Baseline characteristics included age, body mass index (BMI), parity, histological type, and FIGO stage of cervical carcinoma. Chi-square test was applied to compare the nominal data between treatment and control groups. Continuous data were presented as mean ± standard deviation by independent-samples* t*-test and 95% confidence intervals (CI). If *P* < 0.05, data values would be considered as significant difference (two-tailed testing).

## 3. Results

### 3.1. Study Population

**Figure 1:**
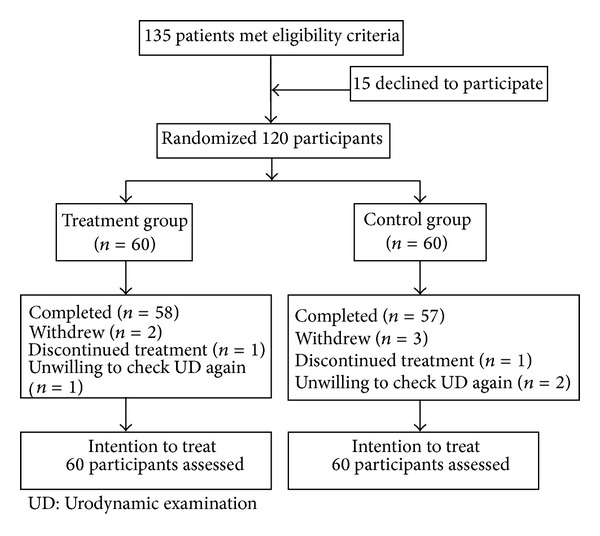
Study flowcharts.

Among 135 patients who met eligibility criteria, 15 of them declined to participate. 120 participants were randomized into treatment group (*n* = 60) and control group (*n* = 60). Treatment group had 2 participants withdrawing and control group had 3 participants withdrawing during the study. The participants who discontinued treatment finished all the outcome measurements, while the participants who were unwilling to check urodynamic examination again on the 30th postoperative day finished all the other evaluations. None of them withdrew because of adverse events (Figure 1).

The mean age was 46.2 ± 7.9 years and the mean BMI was 24.7 ± 3.4. The baseline clinical characteristics of two groups are presented in Table 1 and had no significant differences between each other (*P* > 0.05).

### 3.2. Outcome Measurements

#### 3.2.1. Bladder Function

Table 2 provides a summary of bladder function of the treatment and control groups on 15th and 30th postoperative days. Compared with control group on the same postoperative day, treatment group had better bladder function in terms of maximal cystometric capacity, maximal flow rate, PVR and bladder compliance (*P* < 0.05). The first voiding desire of treatment group was stronger than that of control group (*P* < 0.05). It also shows that bladder function was improved to a certain extent as time went on from 15th postoperative to 30th postoperative day both in treatment group and in control group (*P* < 0.05).

#### 3.2.2. Postoperative Complications

Complications after RH are presented in Table 3. Bladder dysfunction, including sensory loss, incontinence, and urinary retention, was the most common postoperative complication in both groups. Lymphocyst formation ranked second in the incidence rate. The other complications were rare or even had no occurrence within the 30th postoperative day except urinary tract infection.

Compared with control group, treatment group showed significant reduction in sensory loss, incontinence, and urinary retention on 15th and 30th postoperative days (*P* < 0.05). There were no significant differences between two groups in terms of lymphocyst formation and wound infection on 15th and 30th postoperative days (*P* > 0.05). The occurrences of lymphedema and ileus were rare and showed no significant differences during the study. In terms of urinary tract infection, treatment group showed no advantage on the 15th postoperative day compared with control group but had significant difference on the 30th postoperative day (*P* < 0.05).

#### 3.2.3. Adverse Events

No adverse events were higher than grade I according to CTCAE version 4.0 related to acupuncture treatment during the research. Six subjects had local hematoma around acupoints in treatment group, while one subject reported local hematoma in control group. Three subjects in treatment group developed local muscle convulsion and lasted less than 1 minute. All the above events were mild.

## 4. Discussion

This study was a single-blinded, parallel-group, randomized controlled clinical trial. We aimed to evaluate the preventive effects of acupuncture for complications after RH.

The complications after RH result from partial denervation and structural changes during the operation procedure. The parasympathetic fibers, together with hypogastric (sympathetic) nerves to form pelvic plexus, arise from S3-S4 to form the pelvic nerves which may be severed during sectioning of the caudal parts of rectouterine ligament and rectovaginal ligament. Furthermore, part of the pelvic plexus may be removed along with the paravaginal tissue surrounding the upper 1/3 vagina [15]. The radical dissection will interrupt automatic nerve supply to the bladder.

The urodynamic examination is an objective and validated assessment of bladder function, which will be attenuated after RH due to the above impairment [5, 16]. In this study, the urodynamic results showed bladder function in treatment group was better than that in control group on 15th and 30th postoperative days. Bladder dysfunctional disorders in treatment group, including bladder sensory loss, incontinence, and urinary retention, were less than those in control group. Acupuncture could significantly improve postoperative bladder function and reduce bladder dysfunctional disorders. Some other researches also indicated that acupuncture was helpful for recovery of bladder dysfunctional disorders, including urinary incontinence and retention [17]. The possible mechanism for bladder recovery is the nonselective effect of acupuncture stimulation (electrical/mechanical) at S2–S4 where the pelvic splanchnic nerves and pudendal nerve arise [18, 19]. Acupuncture has positive effects on nerve regeneration process and provides an alternative treatment on nerve-injured patients [20, 21]. For the patients after RH, acupuncture may help the impaired nerve restoration and reconstruction which are essential for preventing bladder dysfunction [11, 22]. Another possible mechanism of acupuncture effect for bladder dysfunction lies in the regulation and balance of neurotransmitter, such as catecholamines. A newly released clinical experiment shows that acupuncture at ST36 and SP6 can regulate secretion of catecholamines [23]. Catecholamines have the physiological action to dominate smooth muscle relaxation through *β*-2 receptor and contraction through *α*-1 receptor.

Literature presented that 14.5% patients after RH required urethral catheterization more than 4 weeks [24]. Prolonged catheterization increases the risk of urinary tract infection [25, 26]. In our study, treatment group had significant advantage to reduce urinary tract infection on 30th postoperative day. All the patients with urinary tract infection in control group on 30th postoperative day were accompanied with urinary retention. Acupuncture for the patients after RH helps to prevent urinary tract infection because of its effect in preventing urinary retention on the 30th postoperative day.

The incidence of lymphocyst formation ranks second in complications after RH according to our study and the previous literature [24]. It can lead to lymphedema and infection subsequently due to interruption of efferent pelvic lymphatics [1]. But acupuncture could not reduce lymphocyst formation in this study protocol. It does not necessarily mean that acupuncture therapy does not work. Maybe the length of treatment or other acupuncture interventions should be taken into consideration. Moxibustion, which is a therapeutic tool based on acupuncture theory, may be applicable to prevent or treat lymphocyst formation and lymphedema because of its effect in increasing deep body temperature [27].

The continuous improvements of RH procedure are beneficial to decrease other complications [1]. In terms of ileus, ureterovaginal fistula and wound infection, the incidences were rare and no significant differences were presented between both groups in this study. The later multicenter research and improvement of acupuncture protocol should focus on resolution of bladder dysfunction and lymphocyst formation. It must be said that we should also try to find the stable predictive factors of postoperative complications [28–30], in order to avoid unnecessary acupuncture treatment and attach more importance to the patients who really need it.

## 5. Conclusion

By improving postoperative bladder function, early intervention of acupuncture may provide a valuable alternative method to prevent bladder dysfunctional disorders and urinary tract infection after radical hysterectomy. But acupuncture did not prevent lymphocyst formation according to this protocol.

## Figures and Tables

**Table 1:** Baseline clinical characteristics of treatment and control groups.

	Treatment group (*n* = 60)	Control group (*n* = 60)	*F* value *χ* ^2^	*P* value
Age (years)	46.5 ± 7.7	45.9 ± 8.2	0.646	0.697
Body mass index (kg/m^2^)	24.3 ± 3.4	25.1 ± 3.3	0.141	0.184
Parity	1.55 ± 1.0	1.58 ± 0.96	0.11	0.853
Histological type (*n* (%))				
Squamous cell carcinoma	49 (81.7)	48 (80)	0.054	0.817
Cervical adenocarcinoma	5 (8.3)	4 (6.7)	0.12	0.729
Endometrial cancer	4 (6.7)	5 (8.3)	0.12	0.729
Other	2 (3.3)	3 (5)	0.209	0.648
FIGO stage of cervical carcinoma (*n* (%))				
I A2	17 (28.3)	19 (31.7)	0.159	0.69
I B1	24 (40)	21 (35)	0.32	0.572
II A1	13 (21.7)	11 (18.3)	0.208	0.648
Other	3 (5)	2 (3.3)	0.209	0.648

FIGO: the International Federation of Gynecology and Obstetrics version 2009.

**Table 2:** Postoperative bladder function of the treatment and control groups (mean ± SD, CI).

	15th postoperative day				30th postoperative day			
	Treatment group	Control group	*F* value	*P* value	Treatment group	Control group	*F* value	*P* value
First voiding desire (mL)	190.22 ± 12.77 186.92–193.52	198.52 ± 16.33 194.30–202.74	3.722	0.002	184.88 ± 10.98 182.05–187.72	191.25 ± 14.44 187.52–194.98	2.136	0.008
MCC (mL)	370.92 ± 13.06 367.54–374.29	362.57 ± 13.46 359.09–366.04	0.01	0.001	384.97 ± 15.73 380.90–389.03	372.92 ± 14.46 369.18–376.65	0.534	<0.001
Maximal flow rate (mL/sec)	8.07 ± 1.75 7.62–8.52	6.42 ± 2.40 5.80–7.04	6.573	<0.001	13.52 ± 2.41 12.89–14.14	10.70 ± 3.13 9.89–11.51	6.38	<0.001
PVR (mL)	92.52 ± 10.37 89.84–95.20	100.53 ± 17.28 96.07–105.00	7.058	0.003	86.05 ± 10.16 83.42–88.68	94.85 ± 11.82 91.80–97.90	0.054	<0.001
BC (mL/cmH_2_O)	22.27 ± 2.81 21.54–22.99	20.03 ± 4.01 19.00–21.07	5.237	0.001	28.22 ± 5.59 26.77–29.66	24.32 ± 4.77 23.08–25.55	0.046	<0.001

MCC: Maximal cystometric capacity; PVR: Postvoided residual urine; BC: Bladder compliance.

**Table 3:** Postoperative complications of the treatment and control groups.

	15th postoperative day				30th postoperative day			
	Treatment group	Control group	*χ* ^2^	*P* value	Treatment group	Control group	*χ* ^2^	*P* value
Sensory loss (*n*)	12	22	4.104	0.043	5	14	5.065	0.024
Incontinence (*n*)	4	12	4.615	0.032	2	8	3.927	0.048
Urinary retention (*n*)	6	15	4.675	0.031	1	7	4.821	0.028
Lymphocyst formation (*n*)	5	4	0.12	0.729	3	5	0.536	0.464
Lymphedema (*n*)	0	0			0	1	1.008	0.315
Ileus (*n*)	0	1	1.008	0.315	0	0		
Ureterovaginal fistula (*n*)	0	0			0	0		
Wound infection (*n*)	1	2	0.342	0.559	1	0	1.008	0.315
Urinary tract infection (*n*)	1	1			0	4	4.138	0.042
